# Practical Notes

**Published:** 1895-12

**Authors:** L. C. Bryan

**Affiliations:** Basel, Switzerland


### Practical Notes.

BY L. C. BRYAN, BASEL, SWITZERLAND.

Abstract of paper read at American Dental Association, Aug., 1895.

### NEW MAT GOLD.

This gold has been brought out by Dr. De Trey, and has been given the name of Selila. It is a mat gold of superior qualities. It welds with remarkable facility, and it is said a filling can be made in one-third the time required with any other gold. Dr. Bryan passed around some samples of gold for inspection.

### SPONGE HOLDER WITH MIRROR.

This invention consists of a round mirror soldered to the outside of one point of a pair of pliers. With the pliers a piece of wet sponge is held on the filling or tooth to be ground, and when desired to look at the work with the mirror, it can be done by simply turning the pliers over and thus exposing the face of the mirror. It is used to moisten the corundum or any other grinding wheel, and at the same time protects the tongue and lips. It catches the gold that falls when polishing, and keeps the corundum cold, sharp and clean. The White moistening pads or any other moisture holder may be used with it. Sponges are preferable, as they do not catch in the grinding wheels. Sponges or pads should be kept ready moist in a dish of water, and after use should be preserved in a dish to burn and recover the gold scraps.

After grinding down and burnishing the filling, dip the wet sponge or pad into the polishing powder desired and convey it to the filling and buff or wood wheel or point to be used.

### MIRROR WITH SHELL-SHAPED RIM.

This is very useful when filling the upper teeth, as it conveys the filling material to the cavity and catches all the fragments which may drop during the operation.

It throws light upon the tooth and reflects the operation and the cavity at the same time. Moreover, it is useful in catching chips and old fillings or any other small object removed from the teeth or cavities.

### THE CERVICAL CLAMP

May be described as a bow clamp soldered to a spring cravat-holder. It is very easy to hold, as the fingers and thumb get a good grip of the roughened handle or ends. By pressing the back, the clamp can be made to slide up or down, so as to fasten it at the back. The front bow having been cut, it can be bent in all directions, so that it can be fitted to the most irregular cavity.

